# A new species and a new record of the genus *Cunaxa* von Heyden (Prostigmata, Cunaxidae) from Saudi Arabia, with a key to the world species

**DOI:** 10.3897/BDJ.13.e174602

**Published:** 2025-12-15

**Authors:** Nasreldeen Ahmed Elgoni, Jawwad Hassan Mirza, Fahad Jaber Alatawi

**Affiliations:** 1 Department of Plant Protection, College of Food and Agriculture Sciences, King Saud University, Riyadh, Saudi Arabia Department of Plant Protection, College of Food and Agriculture Sciences, King Saud University Riyadh Saudi Arabia

**Keywords:** Alhasher Mountain, diversity, mites, taxonomy, *
womersleyi
*

## Abstract

**Background:**

The members of the genus *Cunaxa* von Heyden, known as free-living predators, feed on small arthropods and invertebrates in soil. The genus comprises more than 70 described species reported from different regions around the world. In this study, a new species, *C. acaciae* sp. nov., is described and illustrated based on females, and *C. womersleyi* Baker and Hoffmann is reported as a new record to the cunaxid mite fauna of Saudi Arabia.

**New information:**

A new species of the genus *Cunaxa* von Heyden, *C. acaciae* sp. nov., is described and illustrated, based on females collected from soil under *Acacia* sp. (Fabaceae). In addition, *C. womersleyi* Baker and Hoffmann is reported as new to the cunaxid mite fauna of Saudi Arabia. An updated key to the species of the genus *Cunaxa* is provided.

## Introduction

The species of the genus *Cunaxa* von Heyden (Prostigmata, Cunaxidae) are cosmopolitan, free-living predators that feed on small arthropods and invertebrates such as nematodes in soil (Skvarla et al. 2014, Laniecka et al. 2021, Chen et al. 2023). *Cunaxa* is considered amongst the most diverse genera in the subfamily Cunaxinae, represented by more than 70 described species (Skvarla et al. 2014, Kalúz and Ermilov 2023, Khaustov and Khaustov 2024, Mirza et al. 2025). Skvarla et al. (2014) updated the diagnosis of *Cunaxa* and provided a key to 44 species known till that time. Later, Kalúz and Ermilov (2023) provided the key to 68 Cunaxa species. Since then, five additional species have been described: Chen et al. (2023) described two species from China, two species from Russia by Khaustov and Khaustov (2024) and one species from Saudi Arabia (Mirza et al. 2025).

Previously, only seven *Cunaxa* species were reported from Saudi Arabia (SA): *C. arabica* Mirza, Kamran and Alataw; *C. capreolus* (Berlese), *C. clusus* Bashir and Afzal; *C. jatoiensis* Bashir and Afzal; *C. leuros* Bashir, Afzal, Ashfaq, Akbar and Ali; *C. pantabanganensis* Corpuz-Raros and Garcia; and *C. setirostris* (Hermann) (Hermann 1804, Berlese 1887, Corpuz-Raros and Garcia 1995, Bashir and Afzal 2006, Bashir and Afzal 2009, Bashir et al. 2010, Alatawi and Kamran 2017, Mirza et al. 2025). In the present study, a new species, *Cunaxa acaciae* sp. nov., is described and illustrated, based on females collected from the soil under *Acacia* sp (Fabaceae). Additionally, *C. womersleyi* Baker and Hoffmann (Baker and Hoffmann 1948), is reported as new to the cunaxid mite fauna of SA. Subsequently, an updated key to the known species of the genus is provided.

## Materials and methods

Soil debris under *Acacia* sp. was processed through Berlese-Tullgren funnels to collect mites. The specimens were mounted directly in Hoyer’s medium on glass slides under a stereomicroscope (SZX10, Olympus, Tokyo, Japan) and were identified under a phase contrast microscope (DM2500, Leica, Wetzlar, Germany). The species was identified by following the key published by Kalúz and Ermilov (2023) and compared with other species described later. The images of different body parts were captured by using an auto-montage software system (Syncroscopy, Cambridge, UK). These images were used as templates and illustrated by Adobe Illustrator (Adobe System Inc., San Jose, CA, USA). The terminology and setal nomenclature of the venter follows Den Heyer (1981); the dorsal and anal setae follow Skvarla et al. (2014); the palp chaetotaxy follows Khaustov (2020) and the leg chaetotaxy follows Grandjean, overviewed by Norton (1977) and applied to Cunaxidae by Khaustov (2020). The holotype measurements for the morphological features are provided as a single value followed by those of the paratypes in parentheses as ranges. All measurements are given in micrometres (μm). All specimens of new species and new record have been deposited in the King Saud University Museum of Arthropods (KSMA), Acarology section, Department of Plant Protection, College of Food and Agriculture Sciences, King Saud University, Riyadh, Saudi Arabia.

### Abbreviations

*asl*, attenuate solenidion; *bsl*, blunt-ended solenidion; *bbsl*, blunt bulbous solenidion; *eup*, eupathidium; *sts*, simple tactile seta; *dt*, tibial trichobothrium. Tr, trochanters; BFe, basifemora; TFe, telofemora; Ge, genu; Ti, tibia; Ta, tarsus.

## Taxon treatments

### Cunaxa acaciae sp. nov.

406CAEE7-84B4-5D89-A544-24DB523E4279

AE488DA4-9EBC-4D18-A6B3-197538358700

#### Materials

- **Type status:** Holotype. **Occurrence:** catalogNumber: KSMAAS25-Cun-Cun-H; recordedBy: E.M. Khan, N.A. Elgoni and H.M.S. Ali; individualCount: 1; sex: Female; lifeStage: adult; occurrenceID: C3872FF1-4DB0-55BB-8D66-E6D8E0455FEA; **Taxon:** scientificName: Cunaxa acaciae; kingdom: Animalia; phylum: Arthropoda; class: Arachnida; order: Prostigmata; family: Cunaxidae; genus: Cunaxa; **Location:** country: Saudi Arabia; stateProvince: Jazan; locality: Alhasher Mountain; verbatimCoordinates: 17°28.252'N 43°2.095'E; decimalLatitude: 17.47087; decimalLongitude: 43.03491; georeferenceProtocol: GPS; **Identification:** identifiedBy: Nasreldeen Ahmed Elgoni; dateIdentified: 2025; **Event:** samplingProtocol: Soil collection; eventDate: 23/2/2025; habitat: Acacia sp.; **Record Level:** language: en; collectionCode: Mites; basisOfRecord: Slide Mounted Specimen
- **Type status:** Paratype. **Occurrence:** catalogNumber: KSMAAS25-Cun-Cun-P1; recordedBy: E.M. Khan, N.A. Elgoni and H.M.S. Ali; individualCount: 1; sex: Female; lifeStage: adult; occurrenceID: B8FB4949-5C28-590B-BA05-6F1ABF425230; **Taxon:** scientificName: Cunaxa acaciae; kingdom: Animalia; phylum: Arthropoda; class: Arachnida; order: Prostigmata; family: Cunaxidae; genus: Cunaxa; **Location:** country: Saudi Arabia; stateProvince: Jazan; locality: Alhasher Mountain; verbatimCoordinates: 17°28.252'N 43°2.095'E; decimalLatitude: 17.47087; decimalLongitude: 43.03491; georeferenceProtocol: GPS; **Identification:** identifiedBy: Nasreldeen Ahmed Elgoni; dateIdentified: 2025; **Event:** samplingProtocol: Soil collection; eventDate: 23/2/2025; habitat: Acacia sp.; **Record Level:** language: en; collectionCode: Mites; basisOfRecord: Slide Mounted Specimen
- **Type status:** Paratype. **Occurrence:** catalogNumber: KSMAAS25-Cun-Cun-P2; recordedBy: E.M. Khan, N.A. Elgoni and H.M.S. Ali; individualCount: 1; sex: Female; lifeStage: adult; occurrenceID: 88C540C2-0794-5C5C-A83D-D24915B26837; **Taxon:** scientificName: Cunaxa acaciae; kingdom: Animalia; phylum: Arthropoda; class: Arachnida; order: Prostigmata; family: Cunaxidae; genus: Cunaxa; **Location:** country: Saudi Arabia; stateProvince: Jazan; locality: Alhasher Mountain; verbatimCoordinates: 17°28.252'N 43°2.095'E; decimalLatitude: 17.47087; decimalLongitude: 43.03491; georeferenceProtocol: GPS; **Identification:** identifiedBy: Nasreldeen Ahmed Elgoni; dateIdentified: 2025; **Event:** samplingProtocol: Soil collection; eventDate: 23/2/2025; habitat: Acacia sp.; **Record Level:** language: en; collectionCode: Mites; basisOfRecord: Slide Mounted Specimen
- **Type status:** Paratype. **Occurrence:** catalogNumber: KSMAAS25-Cun-Cun-P3; recordedBy: E.M. Khan, N.A. Elgoni and H.M.S. Ali; individualCount: 1; sex: Female; lifeStage: adult; occurrenceID: 1EC49FF6-E208-5E86-818B-0D9E7308CD3B; **Taxon:** scientificName: Cunaxa acaciae; kingdom: Animalia; phylum: Arthropoda; class: Arachnida; order: Prostigmata; family: Cunaxidae; genus: Cunaxa; **Location:** country: Saudi Arabia; stateProvince: Jazan; locality: Alhasher Mountain; verbatimCoordinates: 17°28.252'N 43°2.095'E; decimalLatitude: 17.47087; decimalLongitude: 43.03491; georeferenceProtocol: GPS; **Identification:** identifiedBy: Nasreldeen Ahmed Elgoni; dateIdentified: 2025; **Event:** samplingProtocol: Soil collection; eventDate: 23/2/2025; habitat: Acacia sp.; **Record Level:** language: en; collectionCode: Mites; basisOfRecord: Slide Mounted Specimen
- **Type status:** Paratype. **Occurrence:** catalogNumber: KSMAAS25-Cun-Cun-P4; recordedBy: E.M. Khan, N.A. Elgoni and H.M.S. Ali; individualCount: 1; sex: Female; lifeStage: adult; occurrenceID: 22390B26-2630-554B-BD59-5900B17804D6; **Taxon:** scientificName: Cunaxa acaciae; kingdom: Animalia; phylum: Arthropoda; class: Arachnida; order: Prostigmata; family: Cunaxidae; genus: Cunaxa; **Location:** country: Saudi Arabia; stateProvince: Jazan; locality: Alhasher Mountain; verbatimCoordinates: 17°28.252'N 43°2.095'E; decimalLatitude: 17.47087; decimalLongitude: 43.03491; georeferenceProtocol: GPS; **Identification:** identifiedBy: Nasreldeen Ahmed Elgoni; dateIdentified: 2025; **Event:** samplingProtocol: Soil collection; eventDate: 23/2/2025; habitat: Acacia sp.; **Record Level:** language: en; collectionCode: Mites; basisOfRecord: Slide Mounted Specimen
- **Type status:** Paratype. **Occurrence:** catalogNumber: KSMAAS25-Cun-Cun-P5; recordedBy: E.M. Khan, N.A. Elgoni and H.M.S. Ali; individualCount: 1; sex: Female; lifeStage: adult; occurrenceID: 9DE5BCEA-B5A3-57EB-8802-7A889DD41D9B; **Taxon:** scientificName: Cunaxa acaciae; kingdom: Animalia; phylum: Arthropoda; class: Arachnida; order: Prostigmata; family: Cunaxidae; genus: Cunaxa; **Location:** country: Saudi Arabia; stateProvince: Jazan; locality: Alhasher Mountain; verbatimCoordinates: 17°28.252'N 43°2.095'E; decimalLatitude: 17.47087; decimalLongitude: 43.03491; georeferenceProtocol: GPS; **Identification:** identifiedBy: Nasreldeen Ahmed Elgoni; dateIdentified: 2025; **Event:** samplingProtocol: Soil collection; eventDate: 23/2/2025; habitat: Acacia sp.; **Record Level:** language: en; collectionCode: Mites; basisOfRecord: Slide Mounted Specimen
- **Type status:** Paratype. **Occurrence:** catalogNumber: KSMAAS25-Cun-Cun-P6; recordedBy: E.M. Khan, N.A. Elgoni and H.M.S. Ali; individualCount: 1; sex: Female; lifeStage: adult; occurrenceID: 0D9182E0-12E5-59A5-AC0D-F1E13403EC45; **Taxon:** scientificName: Cunaxa acaciae; kingdom: Animalia; phylum: Arthropoda; class: Arachnida; order: Prostigmata; family: Cunaxidae; genus: Cunaxa; **Location:** country: Saudi Arabia; stateProvince: Jazan; locality: Alhasher Mountain; verbatimCoordinates: 17°28.252'N 43°2.095'E; decimalLatitude: 17.47087; decimalLongitude: 43.03491; georeferenceProtocol: GPS; **Identification:** identifiedBy: Nasreldeen Ahmed Elgoni; dateIdentified: 2025; **Event:** samplingProtocol: Soil collection; eventDate: 23/2/2025; habitat: Acacia sp.; **Record Level:** language: en; collectionCode: Mites; basisOfRecord: Slide Mounted Specimen

#### Description

**Female (n = 7).**

Idiosoma length 464 (428–493); idiosoma width 361 (324–349).

**Figure 1.**
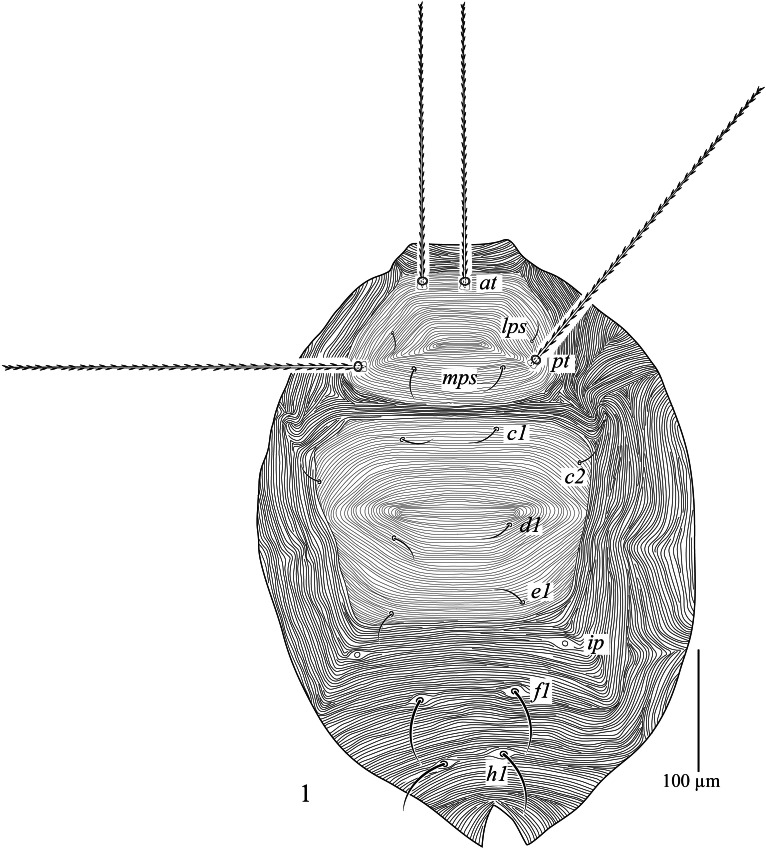
*Cunaxa acaciae* sp. nov., Female, Dorsum. Scale bar 100 μm.

**Dorsum (Fig. 1).** Propodosomal shield with transverse striae medially and diagonal striae laterally, 107 (90–98) long, 172 (171–174) wide, bearing two pairs of trichobothria (*at* and *pt*), two pairs of tactile setae (*lps* and *mps*). Setae *lps* near *pt, pt* 1.24–1.3 times longer than *at, mps* 1.6 times longer than *lps.* Hysterosomal shield 164 (136–140) long, 221 (205–207) wide, bearing four pairs of setae *c_1_*, *c_2_*, *d_1_*, *e_1_*. Setae *f_1_* and *h_1_* on minute sclerotised platelets. Cupules *ip* inserted in integument between setae *e_1_* and *f_1_*. Lateral integument with longitudinal striae, area posterior to hysterosomal shield with transverse striae. Length of setae as follows: *at* 221 (210–213), *pt* 295 (289–296), *lps* 16 (13–15), *mps* 26 (21–22), *c_1_* 28 (23–25), *c_2_* 23(19–20), *d_1_* 26 (20–21), *e_1_* 26 (23–25), *f_1_* 54 (48–53), *h_1_* 56 (53–54). Distance between setae: *at*-*at* 23 (21–23), *pt*-*pt* 134 (123–128), *lps*-*lps* 131 (123–125), *mps*-*mps* 44 (39–44), *lps*-*mps* 46 (41–44), *at*-*lps* 67 (61–64), *pt*-*mps* 43 (38–39), *pt*-*lps* 16 (13–15), *at*-*mps* 71 (62–64), *at*-*pt* 85 (75–79), *c_1_*-*c_1_* 74 (74–75), *c_2_*-*c_2_* 205 (187–189), *d_1_*-*d_1_* 90 (84–89), *e_1_*-*e_1_* 102 (91–96), *f_1_*-*f_1_* 74 (69–74), *h_1_*-*h_1_* 41 (36–39), *c_1_*-*c_2_* 67 (59–60), *c_1_*-*d_1_* 67 (62–66), *c_2_*-*d_1_* 69 (59–62), *d_1_*-*e_1_* 61 (61–62), *e_1_*-*f_1_* 67 (92–92), *f_1_*-*h_1_* 49 (49–54).

**Figure 2.**
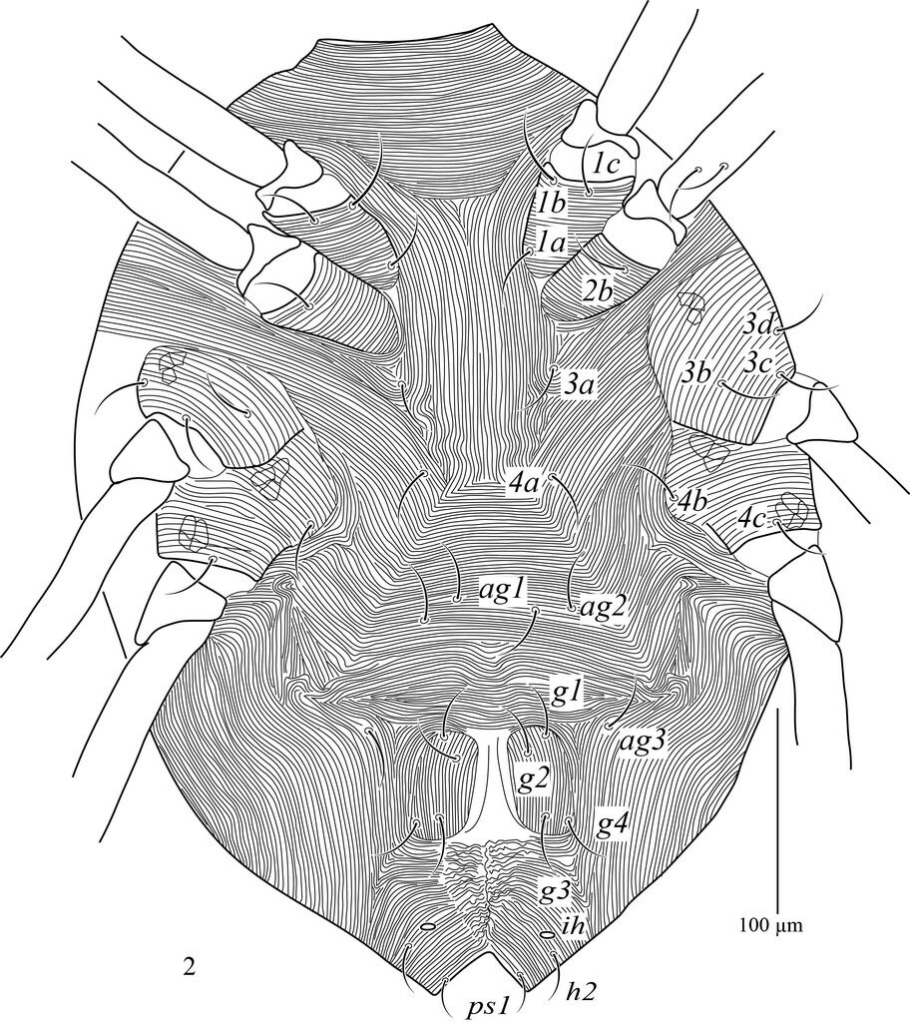
*Cunaxa acaciae* sp. nov., Female, Venter. Scale bar 100 μm.

**Venter (Fig. 2).** Ventral integument striated. Coxae I–II and III–IV contiguous, each with striations; coxae III–IV with additional reticulation elements, intercoxal area with simple longitudinal striae, areas anterior and posterior *ag_3_* with transverse and longitudinal striae, respectively. All ventral setae smooth. Cupules *ih* and setae *h_2_* inserted in integument. Epimeral formula: 3(*1a, 1b, 1c*)-1(*2b*)-3(*3b, 3c, 3d*)-2(*4b, 4c*); two pairs of intercoxal setae *3a* 25 (23–25), *4a* 30 (25–26); three pairs of aggenital setae *ag_1_* 25 (21–26), *ag_2_* 26 (22–24) and *ag_3_* 25 (23–25); genital plates 54 (52–54) long, 33 (31–35) wide, genital plates with four pairs of setae (*g_1_*–*g_4_*), *g_1_* 25 (20–26), *g_2_* 25 (21–25), *g_3_* 33 (21–26) and *g_4_* 31 (25–26); one pair of *h_2_*, 26 (23–26) long; and one pair of pseudanal setae *ps_1_* 16 (15–18) long.

**Figure 3.**
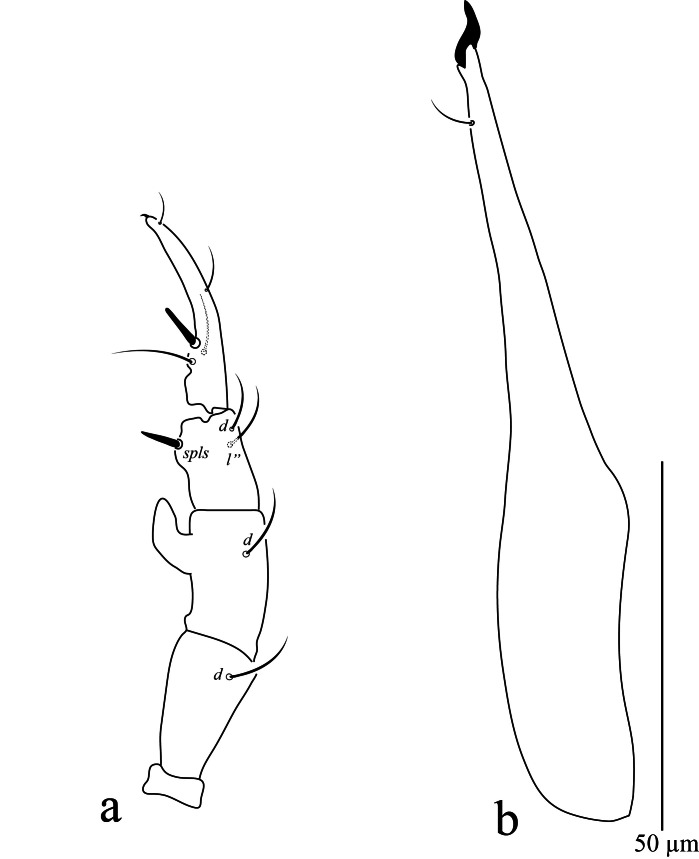
*Cunaxa acaciae* sp. nov., Female, **a**. Palp; **b** Chelicera. Scale bar 50 μm.

**Figure 4.**
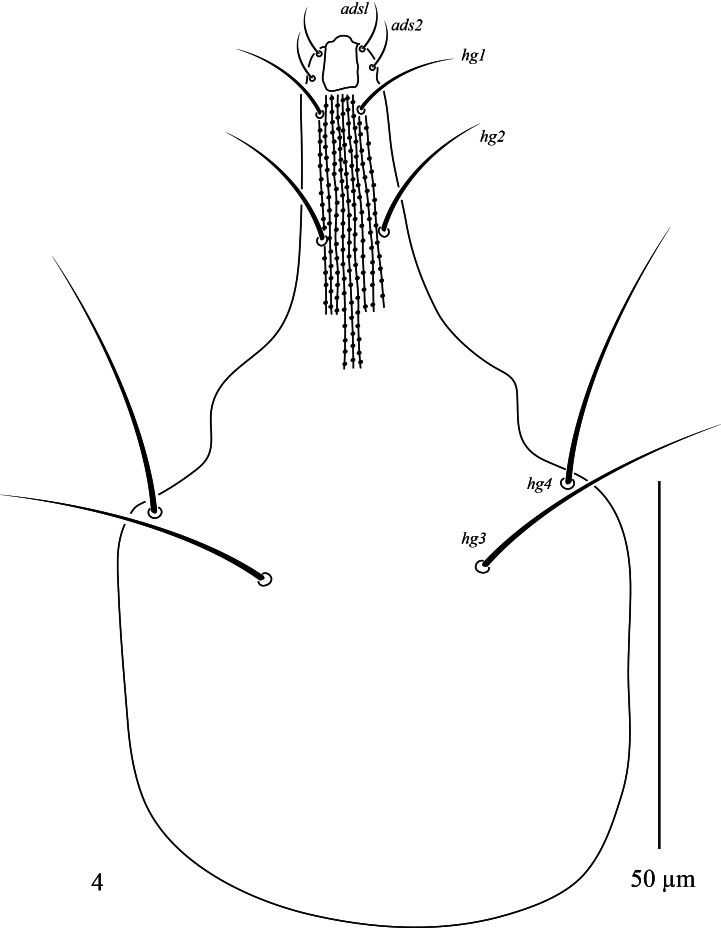
*Cunaxa acaciae* sp. nov., Female, Subcapitulum. Scale bar 50 μm.

**Gnathosoma.** Palp (Fig. 3a) five-segmented, all segments with simple setae. Chaetotaxy: trochanter without setae; basifemur with 1 *sts*; telofemur with 1 dorsal *sts* and hook-shaped apophysis; genu with 2 *sts* and 1 *spls*; tibiotarsus with 4 *sts*, 1 stout rod-like seta and one claw. Chelicera (Fig. 3b). Length of chelicera 108 (102–106). Subcapitulum (Fig. 4) 118 (114–115) long, 65(58–64) wide, with longitudinal pitted striations; four pairs of setae *hg_1_*–*hg_4_* and two pairs of adoral setae *ads_1_* 5 (5–6), *ads_2_* 4 (4–5). Length of hypognathal setae *hg_1_* 16 (15–16), *hg_2_* 15 (15–16), *hg_3_* 21 (17–19) and *hg_4_* 30 (32–33).

**Figure 5.**
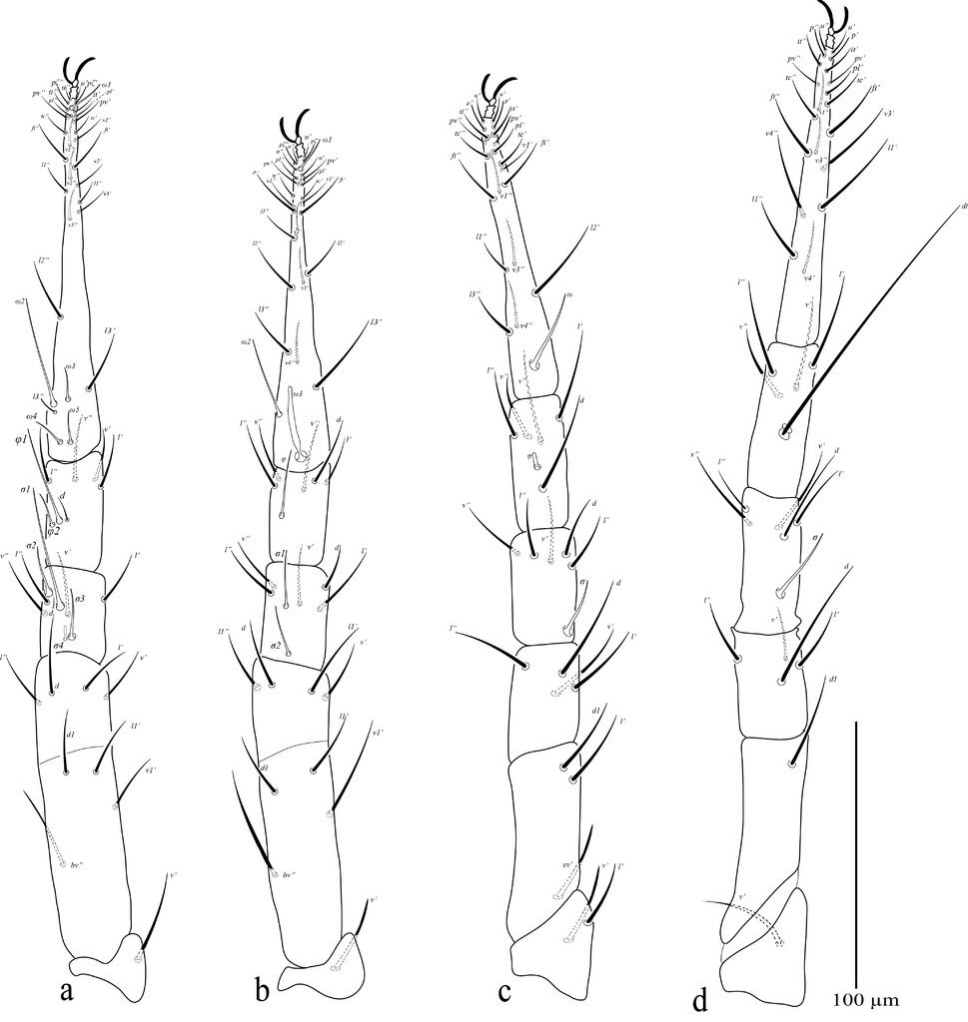
*Cunaxa acaciae* sp. nov., Female, **a** Leg I; **b** Leg II; **c** Leg III; **d** Leg IV. Scale bar 100 μm.

**Legs (Fig. 5).** Leg I 341 (330–343); leg II 331 (328–337); leg III 339 (337–342); leg IV 354 (351–354). Chaetotaxy: Tr I–IV: 1-1-2-1 sts; BFe I–IV: 4 *sts*-4 *sts*-3 *sts*-1 *sts*; TFe I–IV: 4 *sts*-4 *sts*-4 *sts*-4sts; Ge I with 1 *bsl* (*σ4*), 3 *asl* (*σ1*– *3*){1 *asl* (*σ1*), 1 *sts*}, 4 *sts*; Ge II with 2 *asl* (*σ1*–*2*), 5 *sts*; Ge III with 1 *asl* (*σ*), 5 *sts*; Ge IV with 1 *asl* (*σ*), 5 *sts*; Ti I with 2 *asl* (*φ1*–*2*), {1 *asl* (*φ1*), 1 *sts*}, 4 *sts*; Ti II with 1 *asl* (*φ*), 5 *sts*; Ti III with 1 *bsl* (*φ*), 5 *sts*; Ti IV with 1 *dT*, 4 *sts*; Ta I with 4 *asl* (*ω2*–*5*), 1 *dtsl* (*ω1*), 24 sts;Ta II with 1 *bsl* (*ω3*), 1 *dtsl* (*ω1*), 1 *asl* (*ω2*), 24 *sts*; TaIII with 1 *asl* (*ω*), 20 *sts*; TaIV with 20 *sts*.

**Males and immature stages.** Not found.

#### Diagnosis

Propodosomal shield with transverse striae medially and diagonal striae laterally, hysterosomal shield with transverse striae; coxa IV with two setae; area anterior and posterior setae *h_1_* transversely striated.

#### Etymology

The species name (*acaciae*) is derived from the genus *Acacia*, from which the type specimen was collected (collected from soil under *Acacia*).

#### Distribution

##### Remarks

The species *Cunaxa acaciae* sp. nov. is closely related to the *C. capreolus* (Berlese) and *C. bagualensis* Wurlitzer and Ferla (in Wurlitzer et al. (2022)) by having basifemora I, III and IV with 4, 3 and 1 *sts.*, four pairs of setae *c*_1_, *c*_2_, *d*_1_, *e*_1_ on the hysterosomal shield, pedipalp telofemoral apophysis hooked. However, *C. acaciae* sp. nov. can be distinguished from *C. bagualensis* by propodosomal shield with transverse striae medially and diagonal striae laterally, hysterosomal shield with transverse striae (vs. both dorsal shields smooth); integument surrounding propodosomal shield anteriorly and laterally striated (vs. smooth without striae); area anterior and posterior setae *h*_1_ striated (vs. smooth without striae); lateral region of hysterosomal shield covered with longitudinal striae (vs. lateral region of hysterosomal shield with smooth area) in *C. bagualensis*. The new species is distinguished from *C. capreolus* by: propodosomal shield with transverse striae medially and diagonal striae laterally, hysterosomal shield with transverse striae (vs. propodosomal and hysterosomal shields smooth); coxa IV with two setae (vs. with one seta) in *C. capreolus*.

### Cunaxa womersleyi

Baker & Hoffmann, 1948

1BF4F9D5-1146-53FA-8D13-0724AD525E53

#### Materials

- **Type status:** Other material. **Occurrence:** catalogNumber: KSMAAS25-Cun-Cun-1; recordedBy: E.M. Khan, N.A. Elgoni and H.M.S. Ali; individualCount: 1; sex: Female; lifeStage: adult; occurrenceID: DF202137-485D-5933-8988-56DD21B8847B; **Taxon:** scientificName: Cunaxa womersleyi; kingdom: Animalia; phylum: Arthropoda; class: Arachnida; order: Prostigmata; family: Cunaxidae; genus: Cunaxa; **Location:** country: Saudi Arabia; stateProvince: Jazan; locality: Faifa; verbatimCoordinates: 17°15.123'N 43°6.352'E; decimalLatitude: 17.25204; decimalLongitude: 43.10587; georeferenceProtocol: GPS; **Identification:** identifiedBy: Nasreldeen Ahmed Elgoni; dateIdentified: 2025; **Event:** samplingProtocol: Soil collection; eventDate: 23/2/2025; habitat: Soil under Ziziphus sp.; **Record Level:** language: en; collectionCode: Mites; basisOfRecord: Slide Mounted Specimen
- **Type status:** Other material. **Occurrence:** catalogNumber: KSMAAS25-Cun-Cun-2; recordedBy: E.M. Khan, N.A. Elgoni and H.M.S. Ali; individualCount: 1; sex: Female; lifeStage: adult; occurrenceID: B1680152-5BF1-5E95-829D-CEF3E31D8DBA; **Taxon:** scientificName: Cunaxa womersleyi; kingdom: Animalia; phylum: Arthropoda; class: Arachnida; order: Prostigmata; family: Cunaxidae; genus: Cunaxa; **Location:** country: Saudi Arabia; stateProvince: Jazan; locality: Faifa; verbatimCoordinates: 17°15.123'N 43°6.352'E; decimalLatitude: 17.25204; decimalLongitude: 43.10587; georeferenceProtocol: GPS; **Identification:** identifiedBy: Nasreldeen Ahmed Elgoni; dateIdentified: 2025; **Event:** samplingProtocol: Soil collection; eventDate: 23/2/2025; habitat: Soil under Ziziphus sp.; **Record Level:** language: en; collectionCode: Mites; basisOfRecord: Slide Mounted Specimen
- **Type status:** Other material. **Occurrence:** catalogNumber: KSMAAS25-Cun-Cun-3; recordedBy: E.M. Khan, N.A. Elgoni and H.M.S. Ali; individualCount: 1; sex: Female; lifeStage: adult; occurrenceID: 69E2FD80-1DAC-50A7-8A62-4D26C055B14D; **Taxon:** scientificName: Cunaxa womersleyi; kingdom: Animalia; phylum: Arthropoda; class: Arachnida; order: Prostigmata; family: Cunaxidae; genus: Cunaxa; **Location:** country: Saudi Arabia; stateProvince: Jazan; locality: Faifa; verbatimCoordinates: 17°15.123'N 43°6.352'E; decimalLatitude: 17.25204; decimalLongitude: 43.10587; georeferenceProtocol: GPS; **Identification:** identifiedBy: Nasreldeen Ahmed Elgoni; dateIdentified: 2025; **Event:** samplingProtocol: Soil collection; eventDate: 23/2/2025; habitat: Soil under Ziziphus sp.; **Record Level:** language: en; collectionCode: Mites; basisOfRecord: Slide Mounted Specimen
- **Type status:** Other material. **Occurrence:** catalogNumber: KSMAAS25-Cun-Cun-4; recordedBy: E.M. Khan, N.A. Elgoni and H.M.S. Ali; individualCount: 1; sex: Female; lifeStage: adult; occurrenceID: 0A5C3D35-22B3-5B25-94C0-1933DF501408; **Taxon:** scientificName: Cunaxa womersleyi; kingdom: Animalia; phylum: Arthropoda; class: Arachnida; order: Prostigmata; family: Cunaxidae; genus: Cunaxa; **Location:** country: Saudi Arabia; stateProvince: Jazan; locality: Faifa; verbatimCoordinates: 17°15.123'N 43°6.352'E; decimalLatitude: 17.25204; decimalLongitude: 43.10587; georeferenceProtocol: GPS; **Identification:** identifiedBy: Nasreldeen Ahmed Elgoni; dateIdentified: 2025; **Event:** samplingProtocol: Soil collection; eventDate: 23/2/2025; habitat: Soil under Ziziphus sp.; **Record Level:** language: en; collectionCode: Mites; basisOfRecord: Slide Mounted Specimen

#### Description

##### Remarks

The Saudi population of *C. womersleyi* is morphologically similar to the original description of the species (Baker and Hoffmann 1948).

#### Distribution

Mexico (Baker and Hoffmann 1948), Taiwan (Tseng 1980), USA (Smiley 1992), Saudi Arabia (present study).

## Identification Keys

### Key to females of known *Cunaxa* species updated after Kalúz & Ermilov (2023)

**Table:** 

1	Setae *lps* absent	2
–	Setae *lps* present	4
2	Short bulbous blunt solenidion *bbsl* anterior to trichobothrium on tibia IV absent; oval area of thin broken striae around setae *mps* absent	3
–	Short blunt bulbous solenidion *bbsl* anterior to trichobothrium on tibia IV present; oval area of thin broken striae around setae *mps* present; Syria	*C. celineae* Barbar
3	Basifemur IV without seta; tarsus III with 24 *sts*; Crimea, Ukraine	*C. anomala* Khaustov and Kuznetzov
–	Basifemur IV with 1 seta; tarsus III with 22 *sts*; Japan	*C. striata* Shiba
4	Setae *at* short and stubby, less than half the length of *pt*; India	*C. anacardae* Gupta
–	Setae *at* normal, nearly as long as *pt*	5
5	Femur I divided to basifemur and telofemur	6
–	Femur I not divided to basifemur and telofemur; Japan	*C. vulgaris* Shiba
6	Basifemur I with 1 *sts*	7
–	Basifemur I with other number of *sts*	8
7	Basifemora I–IV setal formula 1–2–3–0; telofemora I–IV setal formula 2–2–4–3; India	*C. prinia* Gupta and Paul
–	Basifemora I–IV setal formula 1–1–1–2; telofemora I–IV setal formula 2–2–1–1; India	*C. magniferae* Gupta
8	Basifemora I with 2 *sts*	9
–	Basifemora I with other number of *sts*	11
9	Basifemora II–IV setal formula 3–3–1; Pakistan	*C. dotos* Bashir and Afzal
–	Basifemora II–IV setal formula 2–1–0	10
10	Tibia II with 5 *sts*; Pakistan	*C. mahmoodi* Bashir and Afzal
–	Tibia II with 7 *sts*; Pakistan	*C. okaraensis* Bashir and Afzal
11	Basifemur I with 3 *sts*	12
–	Basifemur I with > 3 *sts*	25
12	Genu I with 3 solenidia	13
–	Genu I with 4 solenidia	19
13	Basifemora I–II 3-4 *sts*; Africa	*C. magoebaensis* Den Heyer
–	Basifemora I–II setal formula other	14
14	Basifemur IV without *sts*; Vietnam	*C. smileyi* Kalúz and Ermilov
–	Basifemur IV with 1 *sts*	15
15	Medial hysterosomal shield absent	16
–	Medial hysterosomal shield present bearing *c_1_*, *c*_2_, *d_1_*, *e_1_*; Crimea, Ukraine	*C. bochkovi* Khaustov and Kuznetsov
16	Propodosomal shield covered with fine papillae; China	*C. papilla* Chen and Jin
–	Propodosomal shield smooth or with papillae near tribothria *at* and *pt*	17
17	Area between setae *d_1_*-*d_1_* with transverse striae; cosmopolitan	*C. setirostris* (Hermann)
–	Area between setae *d_1_*-*d_1_* with longitudinal or V shape striae	18
18	Area between setae *d_1_*-*d_1_* with longitudinal striae; four pairs of genital setae; Vietnam	*C. oblongostriata* Kalúz and Ermilov
–	Area between setae *d_1_*-*d_1_* with V shape striae; five pairs of genital setae; Russia	*C. quinquesetosa* Khaustov and Khaustov
19	Coxae I–IV setal formula 3–1–3–3 *sts*; Zambia	*C. niedbalai* Kazmierski and Laniecka
–	Coxae I–IV with different formula	20
20	Coxae I–IV setal formula 3–2–3–1 sts; dorsal idiosomal shields present; India	*C. eupatoriae* Chinniah and Mohanasundaram
–	Coxae I–IV setal formula 3–1–3–2 sts; dorsal medial hysterosomal shield absent	21
21	Dorsal setae short (*c_1_*-*f_1_*, *c_2_*: 7–10, *h1*: 17); striae between *d_1_–d_1_* and *e_1_– e_1_* longitudinal; the Philippines	*C. mercedesae* Corpuz-Raros and Garcia
–	Dorsal setae longer (19–40); striae between *d_1_–d_1_ and e_1_– e_1_* transversal	22
22	Oval area formed by broken striae around setae *mps* present; Crimea, Ukraine	*C. maculata* Sergeyenko
–	Oval area formed by broken striae around setae *mps* absent	23
23	Genua II proximal solenidion extremely short, its length subequal to the diameter of its alveolus; Crimea, Ukraine	*C. guanotoleranta* Sergeyenko
–	Genua II proximal solenidion long, several times longer than the diameter of its alveolus	24
24	Length of setae *mps* longer than half the distance between their bases; dorsal hysterosomal striae distinctly lobed (= with festoons); Crimea, Ukraine	*C. papuliphora* Sergeyenko
–	Length of setae *mps* shorter or equal to half the distance between their bases; dorsal hysterosomal striae smooth; Crimea, Ukraine, Russia	*C. gordeevae* Sergeyenko
25	Basifemur I with 4 *sts*	26
–	Basifemur I with 5 *sts*	74
26	Basifemur III with 2 *sts*	27
–	Basifemur III with other number of setae	31
27	Palpal telofemoral apophysis uncinate	28
–	Palpal telofemoral apophysis finger-like	29
28	Coxae I–IV setal formula 3–1–3–1 *sts*; medial hysterosomal shield with *c_1_-e_1_, c_2_*; *c_1_* 2–2.5 times longer than setae *d_1_* and *e_1_*; Pakistan	*C. jatoiensis* Bashir and Afzal
–	Coxae I–IV setal formula 2–1–2–1, medial hysterosomal median shield with *c_1_*-*f_1_, c_2_*; *c_1_* twice longer than other dorsal setae; Hungary	*C. subita* Ripka and Laniecka
29	Basifemora I–IV 4–4–2–1 *sts*; medial hysterosomal shield with *c_2_,c_1_-e_1_*; Vietnam	*C. asiatica* Kalúz and Ermilov
–	Basifemora I–IV 4–4–2–0 *sts*; medial hysterosomal shield absent	30
30	Propodosomal shield with broken striae; seta *f_1_* crossing bases of setae *h*_*1*;_ China	*C. striata* Chen and Jin
–	Propodosomal shield smooth; seta *f_1_* not crossing bases of setae *h*_*1*;_ Crimea, Ukraine	*C. yaylensis* Sergeyenko
31	Basifemur III with 3 *sts*	32
–	Basifemora III with 4 *sts*	70
32	Basifemur IV without *sts*	33
–	Basifemur IV with *sts*	34
33	Hysterosomal shield present; genua I–II with 1 *asl*, 4 *sts*; Saudi Arabia	*C. arabica* Mirza, Kamran and Alatawi
–	Hysterosomal shield absent; genua I–II with 2 *asl*, 5 *sts* Crimea, Ukraine	*C. violaphila* Sergeyenko
34	Basifemur IV with 2 or more *sts*	35
–	Basifemur IV with 1 *sts*	36
35	Dorsal propodosomal shield indistinct; apophysis on palpal telofemur cylindrical; setae *c_1_* smooth; Africa	*C. brevicrura* Den Heyer
–	Dorsal propodosomal shield well developed; apophysis on palpal telofemur short, almost cone-like; *c_1_* setose and longer than other hysterosomal setae; Africa	*C. meiringi* Den Heyer
36	Medial hysterosomal shield present (may be barely defined)	37
–	Medial hysterosomal shield absent	60
37	Palpal telofemoral apophysis uncinated (e.g. bent, hook-shaped)	38
–	Palpal telofemoral apophysis present or absent; if present, not uncinated	46
38	Setae *c_1_* on integument	39
–	Setae *c_1_* on medial hysterosomal shield	40
39	Tibia I with 2 *asl*, 4 *sts*; tarsi I–IV with 23–17–16–12 *sts*; Pakistan	*C. nankanaensis* Bashir and Afzal
–	Tibia I with 3 *asl*, 4 *sts*; tarsi I–IV with 17–18–16–14 *sts*; Pakistan	*C. clusus* Bashir and Afzal
40	Setae *f_1_* on medial hysterosomal shield	41
–	Setae *f_1_* off medial hysterosomal shield and on integument	42
41	Setae *f_1_* reaching beyond bases *h_1_*; tibia III with 5 *sts*; tarsi I–IV 12–14–16–12 *sts*; Pakistan	*C. leuros* Bashir, Afzal, Ashfaq, Akbar and Ali
–	Setae *f_1_* not reaching bases *h_1_*; tibia III with 6 *sts*; tarsi I–IV with 14–16–19–14 *sts*; Pakistan	*C. rafiqi* Bashir, Afzal, Ashfaq, Akbar and Ali
42	Coxae I–IV with 3–1–3–2 *sts*	43
–	Coxae I–IV with 3–1–3–1 *sts*	44
43	Propodosomal and hysterosomal shields striated; area anterior and posterior setae *h_1_* striated; Saudi Arabia	*C. acaciae* **sp. nov.**
–	Propodosomal and hysterosomal shields smooth; area anterior and posterior setae *h_1_* smooth; Brazil	*C. bagualensis* Wurlitzer and Ferla
44	Genu I with 2 *asl*, 5 *sts*; tarsi I–IV with 18–19–20–21 *sts*; cosmopolitan	*C. capreolus* (Berlese)
–	Genu I with 3 *asl*; a combination of characters other	45
45	Genu I with 3 *as*l, 3 *sts*; tarsi I–IV with 14–18–17–17 *sts*; Pakistan	*C. pakpatanensis* Bashir and Afzal
–	Genu I with 3 *asl*, 4 *sts*; tarsi I–IV with 12–14–16–16 *sts*; Pakistan	*C. bashiri* Bashir and Afzal
46	Palpal telofemoral apophysis truncated; Africa	*C. carina* Den Heyer
–	Palpal telofemoral apophysis finger-like, not truncated	47
47	Line of small sharp spines on pedipalp tibiotarsus present; Crimea	*C. dentata* Sergeyenko
–	Line of small sharp spines on pedipalp tibiotarsus absent	48
48	Setae *c_2_* on soft integument; Africa	*C. terrula* Den Heyer
–	Medial hysterosomal shield bears *c_2_*	49
49	Medial hysterosomal shield indistinctly defined	50
–	Medial hysterosomal shield distinctly defined	51
50	Setae *f_1_*, *h_1_* smooth; the Philippines	*C. romblonensis* Corpuz-Raros and Garcia
–	Setae *f_1_*, *h_1_* finely setose; Africa	*C. sordwanaensis* Den Heyer
51	Medial hysterosomal shield complemented with *c_1_*, *d_1_*, *c_2_*; Crimea, Ukraine	*C. sudakensis* Khaustov and Kuznetzov
–	Medial hysterosomal shield complemented with *c_1_*–*e_1_*, *c_2_*	52
52	Coxa IV with 1 *sts*	53
–	Coxa IV with 2 *sts*	54
53	Broken striae that form cell-like structures on medial hysterosomal shield present; Thailand	*C. thailandicus* Smiley
–	Broken striae that form cell-like structures on medial hysterosomal shield absent; Mexico, Peru, Hawaii	*C. veracruzana* Baker and Hoffmann
54	Seta *c_1_* longer than all other dorsal setae	55
–	Seta *c_1_* not longer than all other dorsal setae	57
55	Basifemora I–IV 4–4–3–1; Saipan, Saudi Arabia, USA	*C. womersleyi* Baker and Hoffmann
–	Basifemora I–IV 4–4–2–1	56
56	Coxae I–IV 3–1–3–2; propodosomal shield with four pairs of setae: *at, pt*, *mps*, *lps*; Vietnam	*C. sergeyenkoi* Kalúz and Ermilov
–	Coxae I–IV 3–1–3–3; propodosomal shield with three pairs of setae: *pt*, *mps*, *lps*; Hungary	*C. polita* Kazmierski and Ripka
57	Genua I–IV with 3–1–1–1 solenidia	59
–	Genua I–IV with other formula	58
58	Base of palpal telofemoral apophysis with projection; setae *mps* subequal to distance between their bases; Russia	*C. palustris* Khaustov and Khaustov
–	Base of palpal telofemoral apophysis without projection; setae *mps* longer than distance between their bases; Africa	*C. lamberti* Den Heyer
59	Setae *c_1_*-*h_1_* approximately equal in length; Africa	*C. hermanni* Den Heyer
–	Setae *c_1_*-*e_1_* half as long as *f_1_* and *h_1_*; Greece	*C. thessalica* Sionti and Papadoulis
60	Palpal telofemoral apophyse short, uncinate, triangular or stubby	61
–	Palp telofemoral apophysis longer, finger-like	65
61	Palpal telofemoral apophysis uncinate; Pakistan	*C. lodhranensis* Bashir and Afzal
–	Palp telofemoral apophysis other	62
62	Palp telofemora with small sharply terminated triangular apophysis; the Philippines	*C. luzonica* Corpuz-Raros and Garcia
–	Palp telofemoral apophysis cone-like, stubby, bluntly terminated	63
63	Telofemora I–IV 5–5–4–4; each dorsal seta situated on small platelet; the Philippines	*C. minidiscondyla* Corpuz-Raros, Naredo and Garcia
–	Telofemora I–IV 4–4–4–4; dorsal setae not on small platelets	64
64	Genua I–IV setal formula 1 *asl*, 6 *st*s-7–6–6; all dorsal hysterosomal setae short, not reaching bases of following setae; the Philippines	*C. pantabanganensis* Corpuz-Raros and Garcia
–	Genua I–IV setal formula 2 *asl*, 6 *sts*–7–6–6; very long all dorsal hysterosomal setae reach well beyond following setae; USA	*C. mageei* Smiley
65	Coxae I–IV 3–1–4–3; Africa, Zambia	*C. mukuni* Laniecka and Kazmiersk
–	Coxae I–IV 3–1–3–2	66
66	Propodosomal shield smooth; Africa	*C. potchensis* Den Heyer
–	Propodosomal﻿ shield striated	67
67	Setae *f_1_*, *h_1_* spiculate; Africa, Somalia	*C. gazella* (Ewing)
–	Setae *f_1_*, *h_1_* smooth	68
68	Trochanters I–IV 1–1–1–1; Madagascar	*C. corpuzrarosae* Kalúz and Starý
–	Trochanters I–IV 1–1–2–1	69
69	Dorsal propodosomal shield fully striated; setae *c_1_*, *f_1_*, *h_1_* smooth and twice longer than other hysterosomal setae; USA	*C. neogazella* Smiley
–	Dorsal propodosomal shield partially covered by broken striae; *f_1_* smooth and twice longer than other hysterosomal setae; Brazil	*C. butantorum* Wurlitzer and Rocha
70	Palp telofemur with short uncinate apophysis; Pakistan	*C. doxa* Chaudhri
–	Palp telofemur with long cylindrical finger-like apophysis	71
71	Basifemora I–III 4–4–4 *sts*; the Philippines	*C. cogonae* Corpuz-Raros and Garcia
–	Basifemora I–III 4–4–2 *sts*	72
72	Medial hysterosomal shield present; Brazil	*C. jacobdenheyeri* Wurlitzer and Silva
–	Medial hysterosomal shield absent	73
73	Homogenous striation on dorsal central hysterosoma; *at* and *pt* finely setose in proximal part and strongly setose in distal part; Ta I–IV 19–20–17–12 *sts*; Hungary	*C. minuta* Laniecka and Kazmierski
–	Different (wider) striation on dorsal central hysterosoma; *at* and *pt* strongly setose; Ta I–IV 23–23–23–22 sts; Crimea, Ukraine	*C. heterostriata Khaustov and Kuznetsov*
74	Basifemur III with 4 *sts*; tips of *mps* overstepping bases of *pt*; Mexico	*C. evansi* Smiley
–	Basifemur III with 6 *sts*; tips of *mps* not reaching bases of *pt*; Africa	*C. grobleri* Den Heyer

## Discussion

In the present study, *Cunaxa acaciae* sp. nov. is described and illustrated, based on females, and *C. womersleyi* Baker and Hoffmann 1948 is reported as new to the cunaxid mite fauna of SA. An updated key to the species of the genus *Cunaxa* is provided. Additionally, notes on the validity of the three cunaxid species described by Shiba (1984) are added. The absence and presence of the hysterosomal shield were found to be a significant feature to categorise species of the Cunaxa into two distinct groups. However, in the present study, the subdivisions of any kind (subgenera or species group etc.) are not proposed.

In the family Cunaxidae, the subgeneric divisions are not common (Skvarla et al. 2014). In one instance, the genus *Dactyloscirus* Den Heyer Heyer (1978) was once a subgenus of *Scirus* (now a synonym of *Bdella* in Bdellidae Dugès, 1834) described, based on the shape of the palpfemur apophysis. Vitzthum Vitzthum (1931) and Thor and Willmann Thor and Willmann (1941) elevated *Dactyloscirus* to genus level. Although Skvarla et al. Skvarla et al. (2014) have thoroughly reviewed the taxonomy of the family Cunaxidae, it is anticipated that the significance of certain morphological characters to propose taxonomic divisions in species-rich cunaxid genera would be possible in the future.

**Comment on three *Cunaxa* species described by Shiba (1984)**

Shiba (1984) described seven species from Japan, of which two were already known from the country. Amongst the remaining five species from above, three species, i.e. *C. perforata* Shiba, *C. japonica* Shiba and *C. acicularis* Shiba, are of taxonomic concern; especially *C. japonica*, which was re-identified from the previous misidentification of *C. setirostris* (Shiba 1969).

The two species, *C. japonica* and *C. acicularis*, were differentiated in the key couplet, based on the state of reticulation (evident and weak) and the palpal spine (strong and weak), respectively. However, the presence of weak reticulations was only described for *C. japonica*, while the presence of this character could only be assessed, based on the illustration of *C. acicularis* and *C. perforata*. It contradicts the diagnostic character of the genus *Cunaxa*, i.e. the prodorsal shield is never reticulated. Furthermore, the dorsal striation pattern on the integument of the three species (*C. perforate*, *C. japonica* and *C. acicularis*) has been described as “papillae-bearing,” which also contradicts the *Cunaxa* diagnosis.

Two years later, Shiba (1986) mentioned that most of the species described by Shiba (1984) should be transferred to the genus *Rubroscirus* Den Heyer, 1979 (Den Heyer 1979). Interestingly, the author treated the genus *Rubroscirus* as a synonym of *Cunaxa* (Shiba 1984), but did not comment on the validity of the former genus in the 1986 work. Additionally, the diagnosis of *Rubroscirus* states that the prodorsal sensillae (*at* and *pt*) are not densely pilose (Skvarla et al. 2014). In contrast, the *at* and *pt* are described as “closely ciliated” in *C. japonica*, *C. acicularis* and *C. perforata* (Shiba 1984).

It is significantly important to mention that the work of Shiba (1984) and the species described there have not been considered in the more recent publications on *Cunaxa* species (Skvarla et al. 2014, Kalúz and Ermilov 2023). Den Heyer (2011) suggested transferring 13 *Cunaxa* species to *Rubrioscirus* due to the presence of reticulation on the prodorsal shield, but did not comment on the status of these three species.

The distinct diagnostic characters of the *Cunaxa* and *Rubroscirus* question the validity of the three species. It is thereby concluded that the three species are doubtful and are considered species inquirenda. Their genus designation should be confirmed after the type examination.

## Supplementary Material

XML Treatment for Cunaxa acaciae

XML Treatment for Cunaxa womersleyi
